# Perforation of the atrial wall and aortic sinus after closure of an atrial septal defect with an Atriasept occluder: a case report

**DOI:** 10.1186/s13019-021-01441-x

**Published:** 2021-03-25

**Authors:** Zai-Qiang Zhang, Jia-Wang Ding

**Affiliations:** 1 Department of Cardiology, The First College of Clinical Medical Sciences, China Three Gorges University, 183 Yiling Road, Yichang, Hubei Province 443000 People’s Republic of China; 2 Institute of Cardiovascular Diseases, China Three Gorges University, Yichang, Hubei Province 443000 People’s Republic of China

**Keywords:** Atrial septal defect (ASD), Percutaneous intervention, Amplatzer septal occluder (ASO), Atrial and aortic erosion

## Abstract

**Background:**

While the perforation of the atrial wall and aortic sinus after closure of an atrial septal defect (ASD) is rare, it’s life-threatening, with rapid progress and high mortality. To the best of our knowledge, 21 similar cases have been reported since 1976.

**Case presentation:**

We report a 16-year-old male whose atrial septal defect (ASD) was closed using a 12-mm Amplatzer septal occluder (ASO). Atrial wall and aortic sinus perforation occurred 3 months after transcatheter closure, and the patient was discharged after emergency operation. He was discharged on the 12th postoperative day in good overall condition.

**Conclusions:**

With this case report, we want to illustrate that although percutaneous closure of ASD is regarded as a routine procedure, we should not forget the potentially lethal complications, especially cardiac erosion. Therefore, we should carefully evaluate the risk of erosion before surgery, and careful lifelong follow-up is needed.

## Background

In recent years, large-scale epidemiological studies have found that the prevalence of congenital heart disease (CHD) is 9 per 1000 newborns worldwide, and atrial septal defect (ASD) accounts for approximately 6 to 10% of all CHD in children and for 30% of all CHD in adult patients [1]. With the continuous development of the transcatheter approach, the use of the transcatheter device technique has become widely accepted as an alternative therapy to surgery. It has proven to be as effective as surgical ASD closure and offers significant benefits in terms of low invasiveness, shorter hospitalization, and fewer complications. Erosion is a rare but severe complication of transcatheter ASD closure that occurs in 0.2% of patients [2]. Aortic rim deficiency and implantation of an oversized device are risk factors for erosion. Here, we report a case of perforation of the atrial wall and aortic sinus after closure of an atrial septal defect with an Atriasept occluder.

## Case presentation

**Fig. 1:**
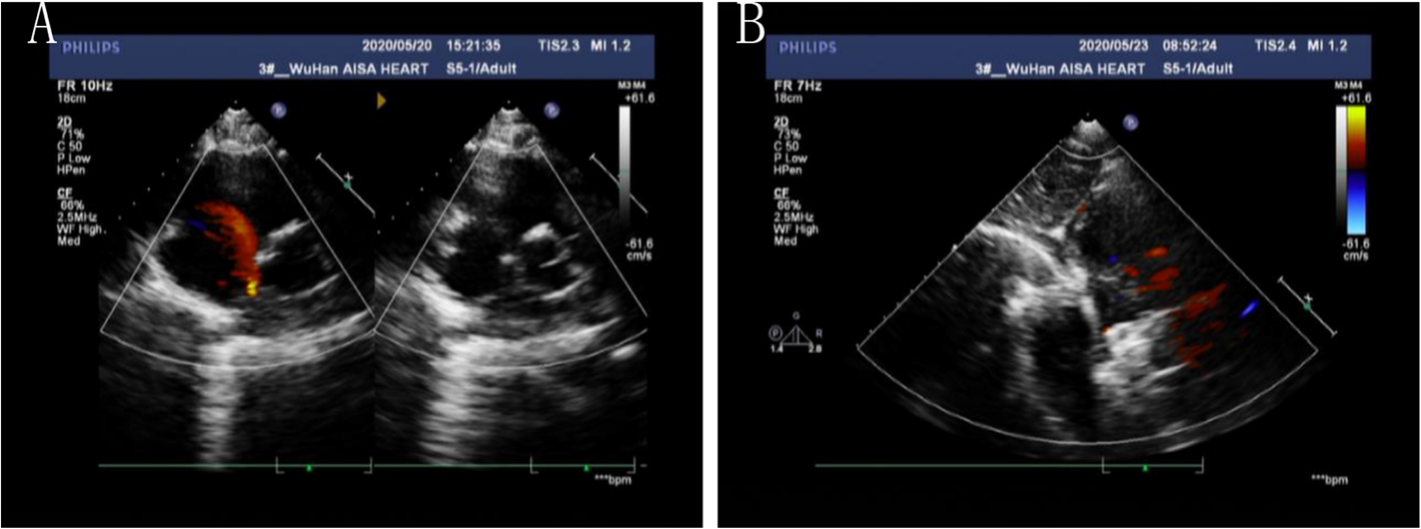
Comparison of TTE before and after transcatheter closure. **a**. Before transcatheter closure, TTE showed continuous interruption of the echo in the central part of the atrial septum. The diameter of the ASD was 0.4 cm, and the shunt signal from the left atrium to the right atrium could be seen in two phases. **b**. After transcatheter closure, TTE showed a strong echo of the occluder in the atrial septum and no shunt at the atrial level

**Fig. 2:**
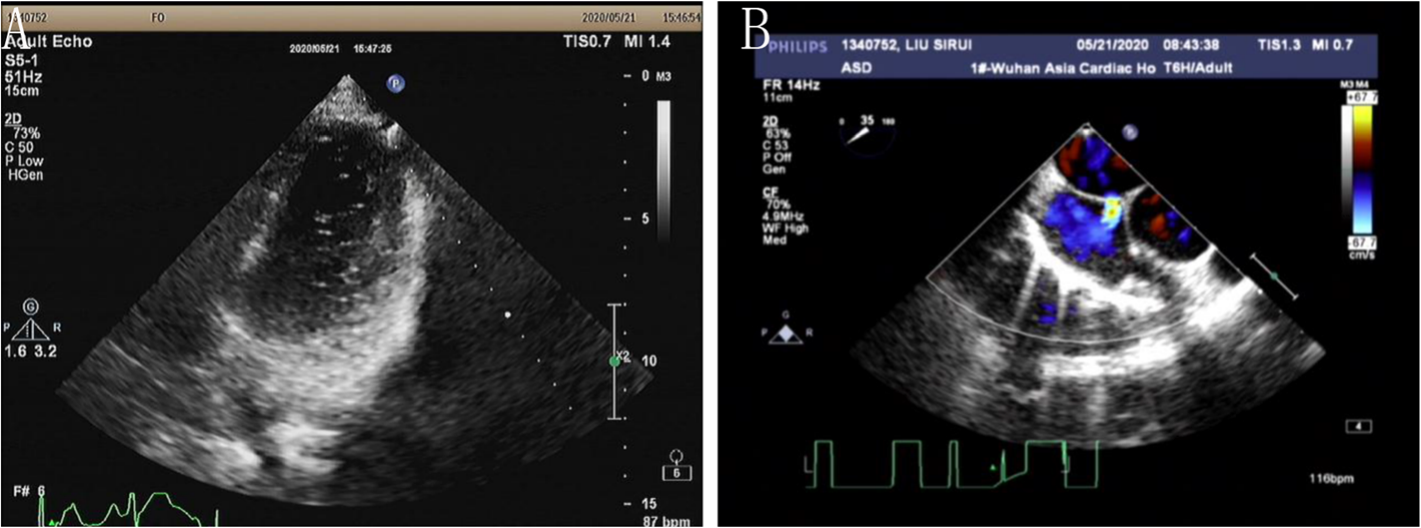
Results for echocardiography of the right heart and TEE before transcatheter closure. **a**. Echocardiography of the right heart showed a large number of right-to-left shunts at the atrial level. **b**. TEE showed that echo interruption was seen in the atrial septum, and a left-to-right shunt signal was seen at the atrial level

**Fig. 3:**
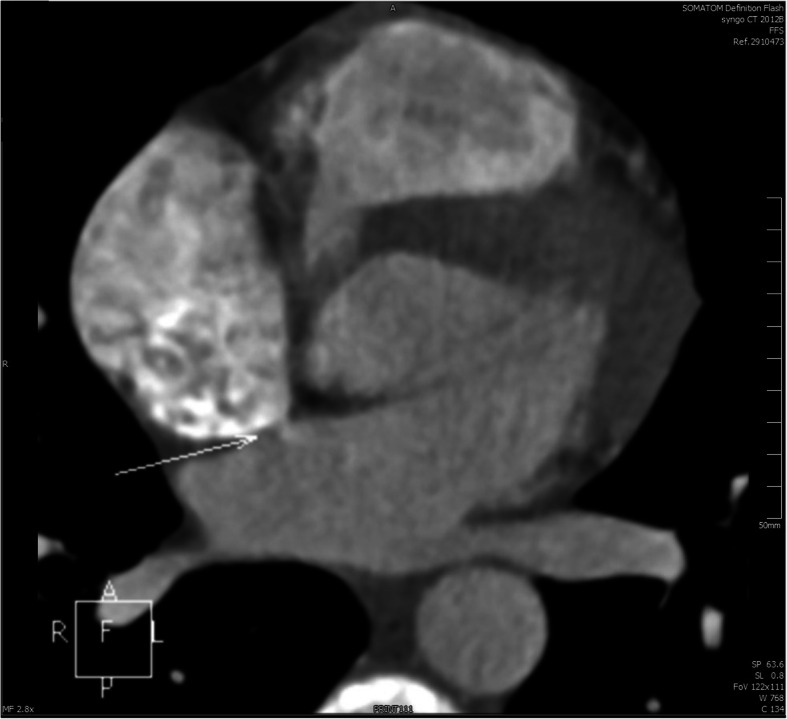
Before transcatheter closure, the cardiac CT showed small ASD

**Fig. 4:**
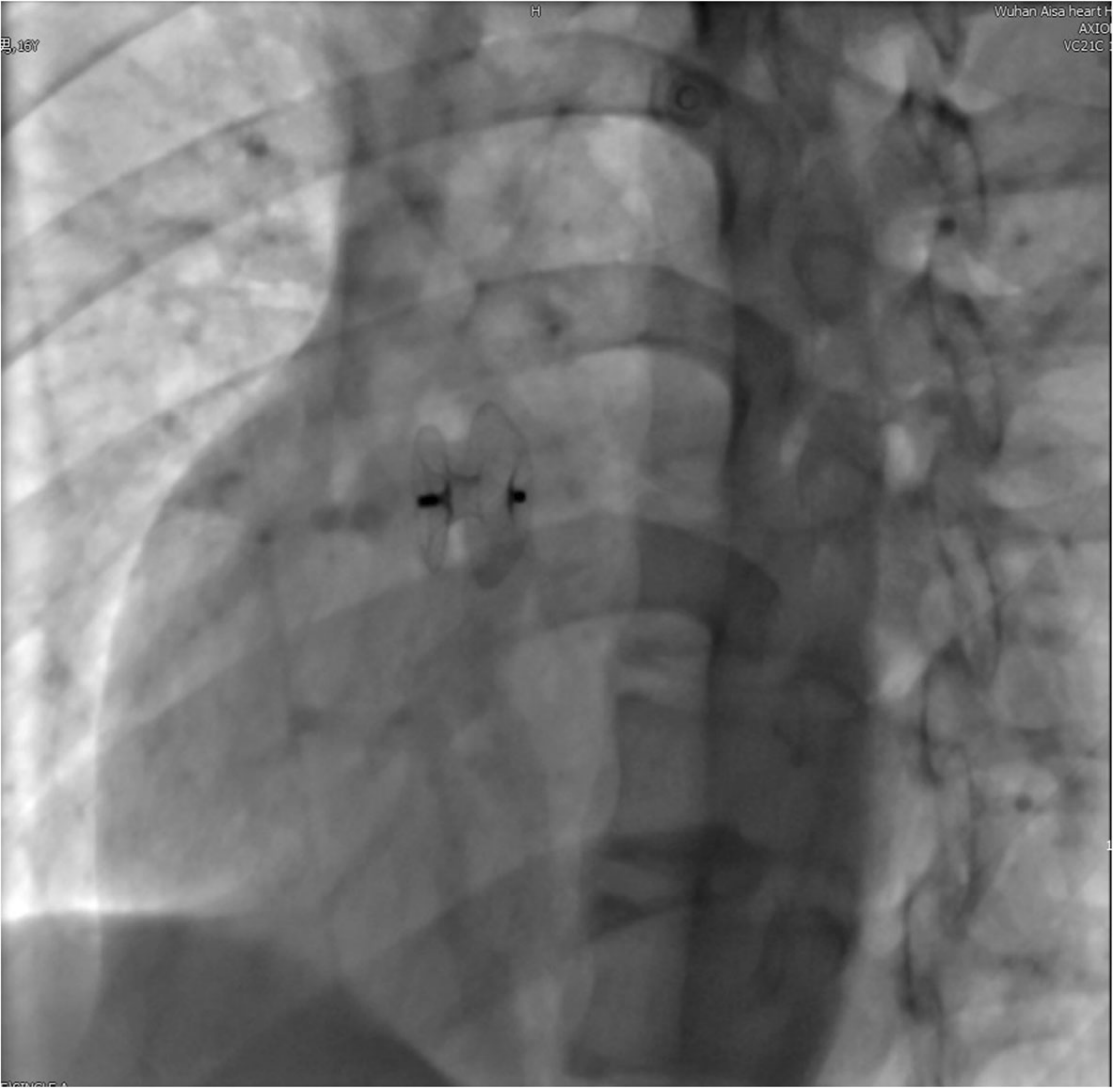
The patient successfully underwent cardiac catheterization and device closure with an ASO

A 16-year-old boy presented with a history of chest tightness for 3 years. Clinical examination favored a diagnosis of an ASD with a left-to-right shunt. Transthoracic echocardiography (TTE) (Fig. 1a) and echocardiography of the right heart (Fig. 2a) confirmed the diagnosis and delineated the morphology of the ASD. He was scheduled for transesophageal echocardiography (TEE) (Fig. 2b), and the ASD measured 0.6 cm. The septal rim was at least 5 mm from the right pulmonary veins, superior vena cava, os of the coronary sinus, and mitral valve. The cardiac Computed tomography (CT) showed a small ASD (Fig. 3). The laboratory examination and electrocardiogram (ECG) showed no abnormalities. The patient underwent cardiac catheterization and device closure with an Amplatzer septal occluder (ASO) (AGA Medical, USA). Balloon sizing was performed under fluoroscopic and TTE guidance. The ASD measured 6 mm, and accordingly, a 12-mm ASO device was selected and deployed successfully across the defect by the conventional technique as described previously. After confirmation of the position and stability of the device on fluoroscopy and TTE, it was released (Fig. 4). The patient underwent a predischarge TTE study on postintervention day 7 (Fig. 1b). The device straddled the ASD well, and there was no obvious shunt on color-flow mapping. No short-term complications occurred, and further follow-up remained uneventful.

**Fig. 5:**
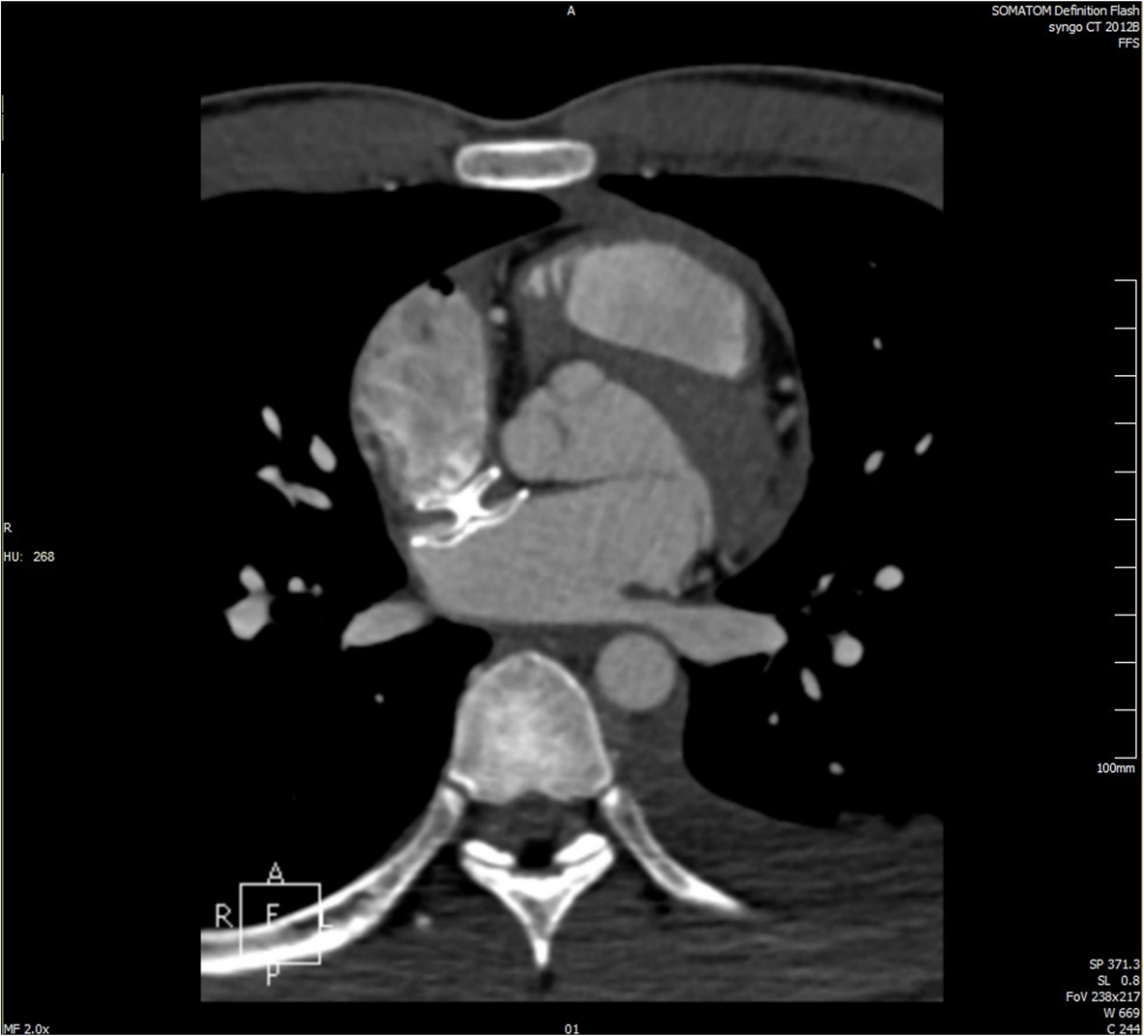
After the patient developed chest pain, cardiac CT showed changes in the atrial septal defect since transcatheter closure and moderate pericardial effusion

Three months later, the patient suddenly developed chest pain that could not be relieved. The ECG obtained at admission showed no abnormality. The laboratory examination results were as follows: blood potassium (K^+^) 3.27 mmol/L; cardiac troponin I (cTn I) 1.67 ng/ml; creatine kinase (CK) 2362 U/L; creatine kinase-MB (CKMB) 6.6 ng/ml; serum creatinine 153 μmol/L; D-dimer 0.79 μg/ml. Blood examination showed leukocytosis. The patient’s blood pressure (BP) was 94/57 mmHg, and his heart rate was 95 bpm. Emergency echocardiography was performed immediately and showed a large amount of pericardial effusion. Cardiac CT showed changes after transcatheter closure of the atrial septal defect and moderate pericardial effusion (Fig. 5). The patient, in shock, underwent urgent operation. Intraoperative examination revealed that the size of the heart was normal, a large amount of bloody effusion was found in the pericardium, about 1-2 mm crevasse was seen in the right atrium roof and noncoronary aortic sinus, and the occluder was well fixed, without displacement or falling off at the atrial septum. We placed a temporary pledgeted suture on the ruptured noncoronary sinus of the ascending aorta to stop the bleeding through a very small perforation. After standard bicaval cannulation, we initiated cardiopulmonary bypass. The right atrium was opened after aortic cross-clamping and antegrade delivery of blood cardioplegia. The ASO was then removed, and a temporary autologous pericardial patch was constructed for the ostial secundum ASD. Finally, the right atrial and noncoronary aortic sinus walls were primarily repaired. The patient was extubated on the first postoperative day, and no neurologic or other major sequelae were seen. He was discharged on the 12th postoperative day in good overall condition. Color Doppler ultrasound showed no pericardial effusion or shunt at the atrial level postoperatively.

## Discussion and conclusions

Percutaneous ASD closure has established itself as a routine intervention due to the effectiveness and safety of the procedure as well as the advantage of a short learning curve. Rupture of the free right atrial wall must be considered a severe complication of percutaneous CHD interventions. This may be a potentially life-threatening complication if it leads to cardiac tamponade. Atrial erosion has been reported in association with Amplatzer septal occluders [3, 4]. According to the data reviewed by Pediatric and Adult Interventional Cardiac Symposium (PICS) 2012 [5], most of the erosion occurred near the aortic root and the top of the atrium: 47 cases involved perforation of the roof of the left atrium (28 cases involved the aorta), 26 cases involved perforation of the roof of the right atrium (22 cases involved the aorta), and 9 cases involved both atria; in 15 cases, the site was unknown. Similarly, in Pics 2012 data [5], approximately 87.6% of erosions occurred in the first year after implantation. The incidence of erosions in children was 57% within 72 h after the operation, and it was 65% in adults after more than 72 h after implantation.

Our case demonstrated perforation of the atrial wall and aortic sinus as a complication of the atrial septal defect closure device 3 months after implantation of a 12-mm ASO. Since erosion occurs several hours or even days after a successful transcatheter closure, it can be concluded that the damage mechanism is not related to a catheterization technique-related complication but rather to a complex interaction between the device and the atrial wall. According to previous experience with occlusion, the base of the aorta is adjacent to the anterosuperior rim or limbus of the defect. In some cases [6], it is the only structure of this boundary. Due to the position of the device against the interatrial septum, the anterosuperior aspect of the device is in constant contact with the aorta during the cardiac cycle [7]. Moreover, due to defects in the aortic rings, especially with the deployment of a significantly oversized device, the edge of the device may elongate the free atrial wall adjacent to the atrial septum. With each cardiac cycle, the movement of the edge of the device near the superior edge leads to a “seesaw” mechanism, resulting in potential erosion of the atrial roof and the adjacent aorta. The result is the development of pericardial hemorrhage and hemodynamic damage. The vulnerability of the atrial wall becomes more exaggerated with oversizing of the device and reduction of the RA cavity after ASD closure [8]. We also found that compared with other atrial septal occluders, AGA occluder material has greater hardness. If placed near the aortic wall, this structure will lead to more rigid contact with the atrial wall and aortic wall, resulting in a higher risk of atrial and aortic erosion.

However, the current analysis of and theory on how device erosion occurs have significant limitations. Therefore, we should pay attention to the following points in future transcatheter closure: first, we should rigorously identify the indications before the operation; second, we must improve transthoracic echocardiography and transesophageal echocardiography before the operation to preoperatively assess the morphology of the ASD with respect to its location, size, shape, and margins and select the appropriate occluder according to the examination results; third, since events are more frequent in the first year, there should be frequent follow-up in the first year (e.g., serial echocardiography at 1 week, 1 month, 3 months, and 6 months) and clinical follow-up annually with less frequent follow-up after the first year [9]; fourth, clinicians must ensure that patients are well informed of the risks/benefits of transcatheter closure of ASD. Further, sufficient communication between clinicians and their patients who have undergone implantation should be occur to educate patients about symptoms without creating fear and anxiety.

To summarize, with this case report, we want to illustrate that although percutaneous closure of ASD is regarded as a routine procedure, we should not forget the potentially lethal complications, especially cardiac erosion. Therefore, we should carefully evaluate the risk of erosion before surgery, and careful lifelong follow-up is needed.

## Data Availability

The datasets of the current study are available from the corresponding author upon reasonable request.

## Abbreviations

ASD: Atrial septal defect
ASO: Amplatzer septal occlude
CHD: Congenital heart disease
TTE: Transthoracic echocardiography
TEE: Transesophageal echocardiography
ECG: Electrocardiogram
CT: Computed tomography
cTn I: Cardiac troponin I
CK: Creatine kinase
CKMB: Creatine kinase-MB
BP: Blood pressure
PICS: Pediatric and Adult Interventional Cardiac Symposium
